# Ocular sarcoidosis: clinical experience and recent pathogenetic and therapeutic advancements

**DOI:** 10.1007/s10792-020-01531-0

**Published:** 2020-08-01

**Authors:** Rosanna Dammacco, Jyotirmay Biswas, Tero T. Kivelä, Francesco Alfredo Zito, Patrizia Leone, Alberto Mavilio, Dario Sisto, Giovanni Alessio, Franco Dammacco

**Affiliations:** 1 Department of Ophthalmology and Neuroscience, University of Bari “Aldo Moro”, Medical School, Bari, Italy; 2 Department of Uveitis and Ocular Pathology, Sankara Nethralaya, Chennai, India; 3 Department of Ophthalmology, University of Helsinki, Helsinki, Finland; 4 Pathology Department, IRCCS-Istituto Tumori ‘Giovanni Paolo II’, Bari, Italy; 5 Department of Biomedical Sciences and Human Oncology, University of Bari “Aldo Moro”, Medical School Polyclinic, Piazza Giulio Cesare, 11, 70124 Bari, Italy; 6 Social Health District, Glaucoma Center, Azienda Sanitaria Locale, Brindisi, Italy

**Keywords:** Angiotensin-converting enzyme, Bronchoalveolar lavage, Ocular sarcoidosis, T-lymphocyte, Uveitis

## Abstract

**Purpose:**

To describe the ocular manifestations in a cohort of patients with systemic sarcoidosis (SS). Recent advances in the pathophysiology, diagnosis, and therapy of SS are also discussed.

**Methods:**

Data from 115 Italian patients diagnosed between 2005 and 2016 were retrospectively reviewed. All but the first 17 patients underwent a comprehensive ophthalmologic examination. The diagnosis was based on clinical features, the demonstration of non-caseating granulomas in biopsies from involved organs, and multiple imaging techniques. Data on broncho-alveolar lavage fluid analysis, calcemia, calciuria, serum angiotensin-converting enzyme levels and soluble interleukin-2 receptor levels were retrieved when available.

**Results:**

Ocular involvement, detected in 33 patients (28.7%), was bilateral in 29 (87.9%) and the presenting feature in 13 (39.4%). Anterior uveitis was diagnosed in 12 patients (36.4%), Löfgren syndrome and uveoparotid fever in one patient each (3%), intermediate uveitis in 3 patients (9.1%), posterior uveitis in 7 (21.2%), and panuveitis in 9 (27.3%). First-line therapy consisted of corticosteroids, administered as eyedrops (10 patients), sub-Tenon’s injections (1 patient), intravitreal implants (9 patients), or systemically (23 patients). Second-line therapy consisted of steroid-sparing immunosuppressants, including methotrexate (10 patients) and azathioprine (10 patients). Based on pathogenetic indications that tumor necrosis factor (TNF)-α is a central mediator of granuloma formation, adalimumab, targeting TNF-α, was employed in 6 patients as a third-line agent for severe/refractory chronic sarcoidosis.

**Conclusion:**

Uveitis of protean type, onset, duration, and course remains the most frequent ocular manifestation of SS. Diagnostic and therapeutic advancements have remarkably improved the overall visual prognosis. An ophthalmologist should be a constant component in the multidisciplinary approach to the treatment of this often challenging but intriguing disease.

## Introduction

Sarcoidosis is a chronic, multisystem, non-caseating granulomatous disease of unknown etiology. Its clinical course, natural history, and outcome are remarkably variable, as in approximately one-third of patients the disease has a tendency to wax and wane. Although sarcoidosis differs in patients of different ethnicities in terms of its clinical variability and visual outcome [1, 2], among those in whom clinical expression is characterized by acute onset with erythema nodosum and/or asymptomatic bilateral hilar lymphadenopathy the course is usually self-limiting and spontaneous resolution, mostly within the first 2–3 years after the diagnosis, occurs in one-third to half of the patients [3, 4]. However, those with an insidious onset and slow progression will eventually develop a multi-systemic disorder that includes lung involvement and multiple extra-pulmonary lesions [5].

Sarcoidosis is a major cause of ocular inflammation. The prevalence of ocular manifestations in patients with systemic sarcoidosis (SS) has been reported as 12.9% [6], 23% [7], 26% [8], and up to 79.2% [9]. However, given the strikingly protean spectrum of clinical manifestations, the lower rates may reflect, in addition to true geographic and ethnic variations, a possibly missed diagnosis in patients with mild ocular signs and symptoms. Ocular manifestations may be the presenting sign of sarcoidosis in 11–30% of patients [10, 11] who will later develop extra-ocular disease, but ocular findings may be recognized at any time during the course of SS [12, 13].

Although all segments of the eye and its adnexa can be involved, the most common sarcoidosis-related ocular disease is uveitis that may result in permanent visual impairment if not timely diagnosed and properly treated [14–16]. However, across the entire spectrum of uveitides (caused by several systemic diseases), the proportion of patients with sarcoid uveitis is variably reported as 2.4% [17], 3% [18], 6.4% [19], and up to 15% [20], again reflecting ethnicity, geography, and diagnostic challenges.

The purpose of this study was to describe the ocular manifestations in a cohort of patients with chronic SS who were diagnosed, treated and followed-up in a cooperative interdisciplinary approach.

## Materials and methods

This is a retrospective, cross-sectional, observational analysis of the medical records of patients who sometime between 2005 and 2016 were examined at the university hospital of the University of Bari, Italy. The population of this cohort study consisted of 115 Caucasian patients from Apulia and other regions of southern Italy. The study was the result of a collaboration between the hospital’s Ophthalmology and Internal Medicine departments. Prior to the study period, 17 patients had been diagnosed with SS in the Internal Medicine department but had not reported visual disturbances, such that an ophthalmological examination was not requested by the examining physician. Later, two patients in whom ocular manifestations were the presenting feature of sarcoidosis underwent a thorough (ocular and extra-ocular) investigation, which led to our decision that all subsequent SS patients should undergo both an ophthalmological and a general internal medicine examination, whether or not they complained of ocular symptoms. Our study population of 115 patients thus consisted of the17 initially examined patients, the two patients examined thereafter and 96 subsequent patients, all of whom received a thorough examination to achieve a definite diagnosis of SS and establish the extent of organ involvement.

In addition, to provide a comprehensive description of ocular sarcoidosis, collaborations were conducted with the Department of Uveitis and Ocular Pathology, Sankara Nethralaya, Chennai, India, and the Department of Ophthalmology, Helsinki University Hospital, Finland. Both institutions contributed to the design and interpretation of the study and provided representative images of sarcoid uveitis.

The University’s Institutional Review Board approved the study and, given the retrospective nature of the study, which was based on a case records review, waived the need for patients’ written informed consent to study enrollment.

Since no widely accepted gold standard test is available for the diagnosis of sarcoidosis, in our cohort of patients SS was diagnosed on the basis of compatible clinical features, the demonstration of non-caseating granulomas in biopsies of involved organs, and typical radiological findings. All patients received a complete physical examination and work-up in order to assess the baseline extent and severity of the disease as well as the clinical phenotype.

Pulmonary sarcoidosis was confirmed by mediastinoscopy in 61 patients. Endobronchial ultrasound-guided transbronchial fine-needle aspiration of lymph nodes in the hilar, mediastinal or both locations was performed in 47 patients with radiological evidence of pulmonary involvement (41%). Rapid on-site cytological assessment led to the diagnosis in most cases, with additional lymph nodes and/or transbronchial lung biopsies performed when the diagnosis was still uncertain. Broncho-alveolar lavage (BAL) fluid was examined in 55 patients. Elevated cellularity (> 15% lymphocytes) and a CD4/CD8 ratio > 3.5 have a high specificity (93–96%) but a low sensitivity (53–59%) for sarcoidosis [21].

Eighty-four biopsies were carried out in 78 of the 115 patients (68%). The biopsy sites were lymph nodes (*n* = 34 patients), liver (*n* = 19), lymph nodes plus liver (*n* = 2), bronchial mucosa (*n* = 13), skin (*n* = 9), lacrimal gland (*n* = 3), skin plus lacrimal gland (*n* = 1), conjunctiva (*n* = 2), and lacrimal gland plus conjunctiva (*n* = 1). Second biopsies were performed when the results of the first were doubtful or inconclusive. ^18^F-fluorodeoxyglucose positron emission tomography in combination with a computed tomography scan (^18^F-FDG PET/CT) was used to detect occult sites of disease and to pinpoint the organs more suitable for diagnostic biopsy [22, 23].

Serum levels of angiotensin-converting enzyme (ACE), measured by a spectrophotometric kinetic assay, were available for 89 patients, none of whom was taking ACE inhibitors at the time of examination. In a 2016 report, the measurement of soluble interleukin-2 receptor (sIL-2R) was recommended as a useful screening marker for sarcoidosis in patients with uveitis of any type [24]. Since the patients in our study were enrolled between 2005 and 2016, only in the 11 patients with a more recent diagnosis were sIL-2R levels measured by an enzyme-linked immunosorbent assay (ELISA). Three of those patients had ocular involvement.

Serum and urine calcium concentrations, measured using a colorimetric method, were retrieved in 95 patients (including all 33 with ocular sarcoidosis) and in 78 patients (including 27 with ocular sarcoidosis), respectively. Hypercalcemia was defined as a calcium level > 11 mg/dL, and hypercalciuria as a urinary calcium excretion > 320 mg in a 24-h urine specimen.

Serum lysozyme levels were reported in the hospital files of 49 patients. However, this marker is a poorly reliable feature of sarcoidosis, as increases have been detected in 25–26% of patients receiving corticosteroids (CS) at the time of diagnosis [25, 26]. Therefore, serum lysozyme data were not considered further in our study.

All patients underwent a comprehensive ophthalmologic examination, including complete biomicroscopic assessment, intraocular pressure (IOP), visual acuity, pupillary reaction, ocular motility, visual field testing, ophthalmoscopy with fluorescein or indocyanine green angiography, fundus autofluorescence imaging, and optical coherence tomography. Additional tests were performed as requested, both at the time of diagnosis and at variable intervals during follow-up.

Categorical data are reported as the number and percentage. The data of independent groups were compared using Fisher’s exact test or a Chi-squared as appropriate. Mean and standard deviation were used to assess patient age by group; the data were compared using a t-test for independent samples. Sex distributions in the groups were compared using a Chi-squared test. A *p* value < 0.05 was considered significant for all tests. All of the statistical analyses were performed using the R-software, V.3.1.1 (https://www.r-project.org).

Diagnostic procedures were carried out in accordance with the ethical standards of the University of Bari Medical School and conformed with the tenets of the 1964 Helsinki Declaration and its later amendments.

## Results

The results of the diagnostic procedures carried out in our patients with ocular sarcoidosis are summarized in Table 1. Serum levels of ACE were increased in the large majority, although the specificity and sensitivity of a high ACE level for the diagnosis of sarcoidosis are only 90% and 41%, respectively [27]. sIL-2R levels, measured in the serum samples of only 11 out of the 115 patients with SS, were elevated in 8 of them (72.7%), including 3 patients with ocular sarcoidosis. Although sIL2-receptor determinations are more reliable (94% specificity and 98% sensitivity) than the serum ACE level in detecting SS [24], the small number of sera examined in our study prevented an adequate analysis.

**Table 1:** Summary of the relevant diagnostic procedures carried out in 33 patients with ocular sarcoidosis

Angiotensin-converting enzyme	
Increased levels (> 82 U/L)	25/29 (86%)
Not performed	4/33 (12%)
Soluble interleukin-2 receptor	
Increased levels (> 639 U/mL)	3/3 (100%)
Not performed	30/33 (91%)
Calcemia	
Normal levels (9–11 mg/dL)	28/33 (85%)
Increased levels	5/33 (15%)
Calciuria	
Normal levels (31–320 mg/24 h)	16/27 (59.3%)
Increased levels	11/27 (40.7%)
Biopsy site (no. of patients)	
Lymph node	12
Bronchial mucosa	7
Liver	3
Skin	3
Lacrimal gland	2
Skin plus lacrimal gland	2
Conjunctiva	2
Lacrimal gland plus conjunctiva	1
No biopsy performed	1/33 (3%)
Bronchoalveolar lavage fluid	
Cells/mm^3^ mean (SD)	287 (± 145)
Lymphocytes %, mean (SD)	32 (± 29)
CD4 + T-cells > 15%	11/20 (55%)
CD4/CD8 ratio > 3.5	16/20 (80%)
Not performed	13/33 (39%)
Chest computed tomography scan	
No remarkable findings	4/28 (14%)
Hilar adenopathy (HA) and/or mediastinal adenopathy (MA)	10/28 (36%)
Parenchymal involvement (PI)	6/28 (21%)
Variable combinations of HA/MA/PI	8/28 (29%)
Not performed	5/33 (15%)
^18^F-FDG PET/CT	
Active sarcoidosis involvement:	11/16 (69%)
Thorax only	7/11 (64%)
Isolated extrathoracic	1/11 (9%)
Both thoracic and extrathoracic	3/11 (27%)
Not performed	17/33 (51%)

Hypercalcemia was detected at diagnosis in 5 (3 males and 2 females) out of the 33 patients tested (15%), and hypercalciuria in 11 (5 males and 6 females) of the 27 patients tested (41%), with the highest levels of urinary calcium occurring in 3 patients with severe active sarcoidosis. The administration of CS induced a prompt reversal of the metabolic defect (data not shown).

**Fig. 1:**
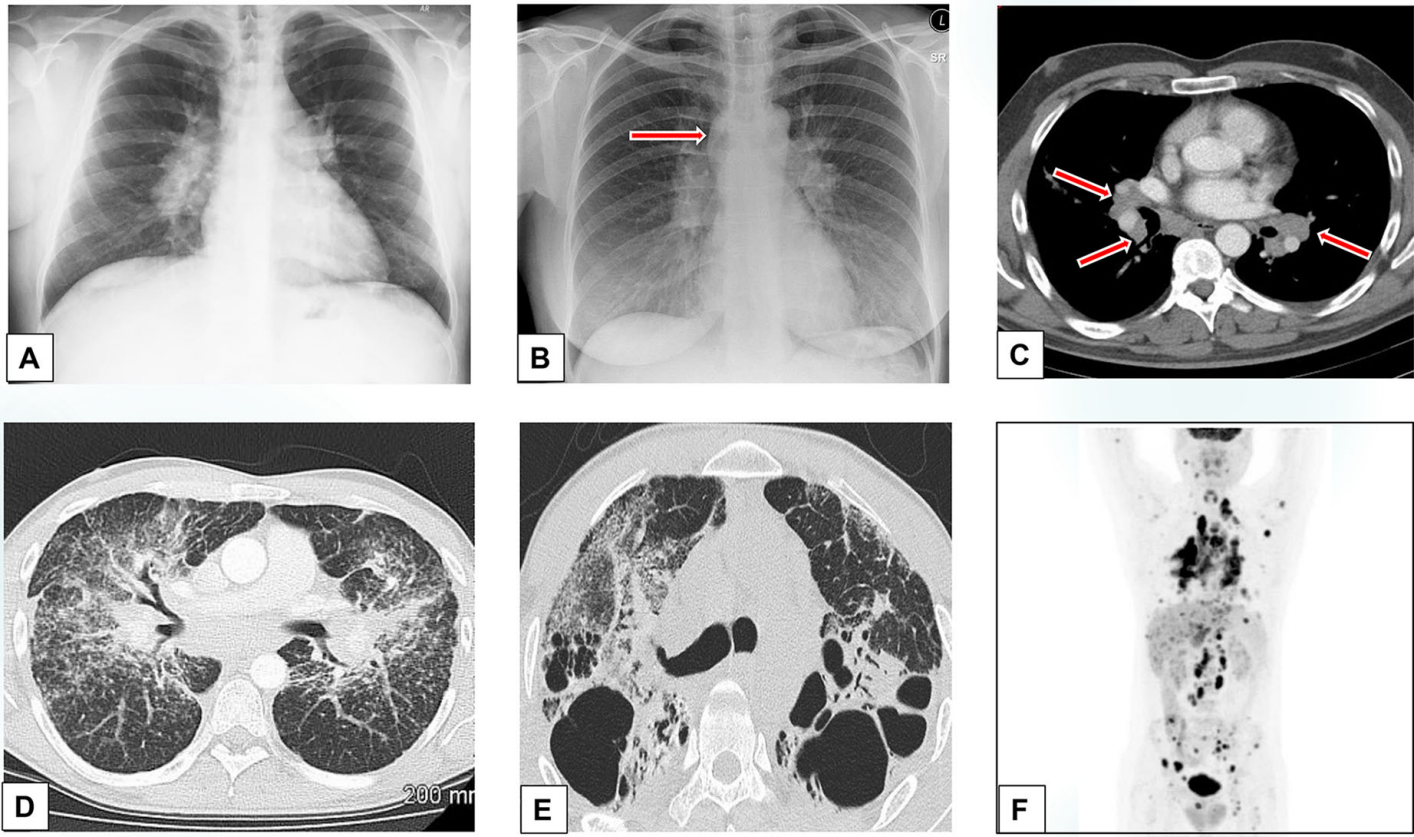
Radiographic patterns seen in the thorax of patients with systemic (including ophthalmic) sarcoidosis. **a** Bilateral hilar lymphadenopathy (BHL) with polycyclic outlines. **b** In another patient, BHL is associated with mediastinal enlargement along the right para-tracheal boundary (arrow). **c** Axial contrast computed tomography (CT) image shows typical BHL (arrows). Lymph nodes with a density lower than that of the vasa are disposed around the bronchi and pulmonary arteries. **d** Micronodules a few millimeters in diameter tend to form granulomatous conglomerates that surround the broncho-vascular peduncles. Lung involvement extends along the broncho-vascular axis, from the hila toward the periphery. Note the diffuse distribution in both lung fields of lung nodules with a typical peri-lymphatic distribution and thickening of the pleural surfaces. **e** Advanced-stage pulmonary sarcoidosis. Fibrosis is typically localized in the hilar and peri-hilar districts and in the upper lobes, with large cystic spaces. The hila are stretched upwards. **f** ^18^F-FDG-PET/CT shows hypermetabolism in many organs and a clear prevalence in both lungs

Pulmonary manifestations seen on chest X-rays and chest CT scan were the most common clinical features, with the findings ranging in severity from isolated bilateral hilar lymphadenopathy (stage 1, Fig. 1a–c) to pulmonary nodules scattered in both lung fields with a perilymphatic distribution and ground-glass parenchymal opacities. Further disease progression resulted in fibrosis, mostly localized in the hilar and peri-hilar regions but also visible in the upper lobes (stage 4, Fig. 1d, e). In selected patients, ^18^F-FDG/PET was employed to locate the most suitable and accessible biopsy site (Fig. 1f) [22].

**Fig. 2:**
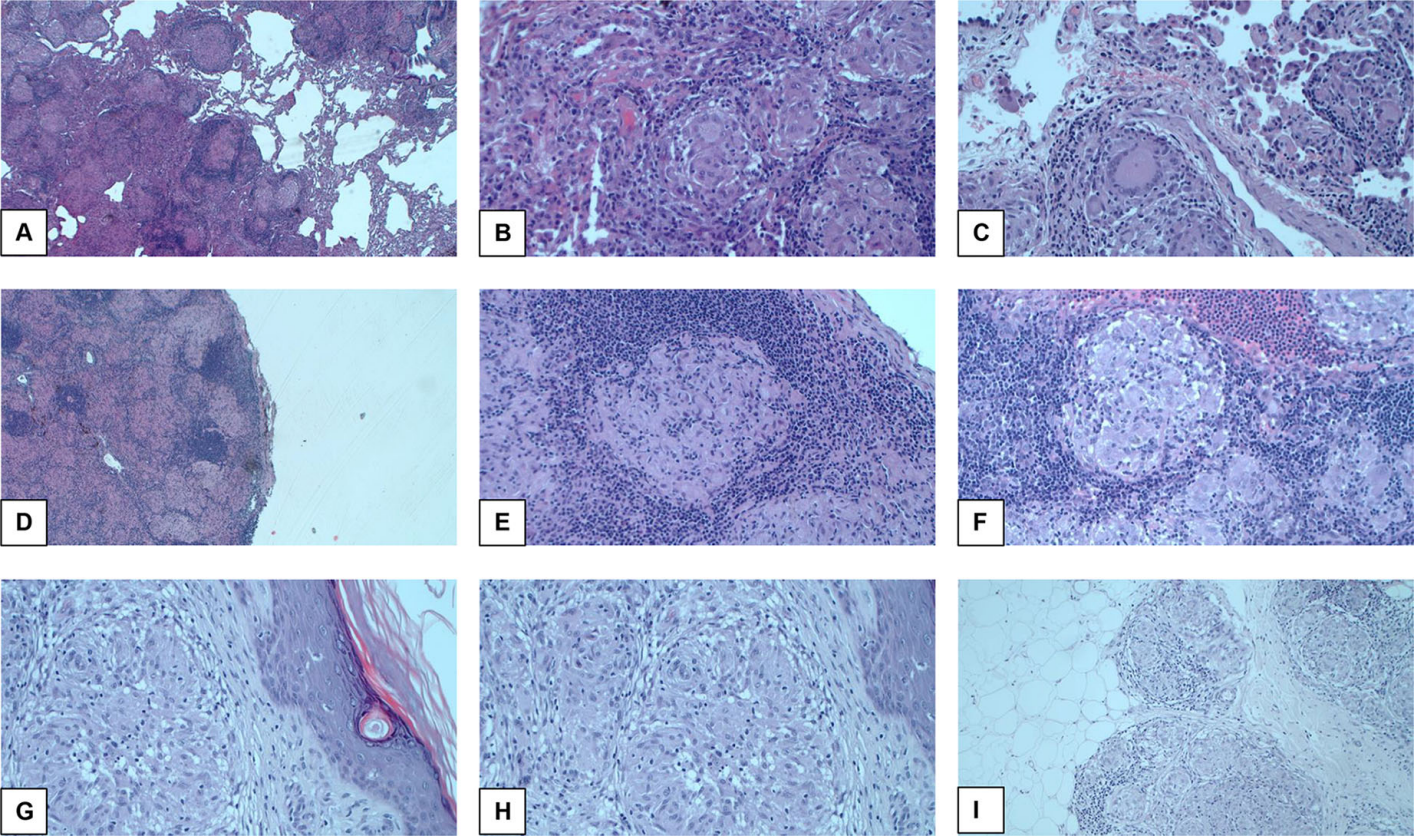
Typical histological features of sarcoidosis. **a–c** Pulmonary sarcoidosis. **a** Non-necrotizing granulomas are distributed along broncho-vascular bundles (hematoxylin–eosin [HE], × 20). **b** Several non-confluent granulomas with tightly packed epithelioid cells and Langhans multinucleated giant cells, surrounded by a sparse rim of lymphocytes (HE, × 200). **c** A well-defined granuloma adjacent to the vessel wall (HE, × 400). D–F: Lymph node sarcoidosis. **d** The nodal architecture is completely replaced by densely packed granulomas (HE, × 20). **e** and **f** Non-necrotizing granulomas consisting of epithelioid histiocytic cells (more clearly recognizable in **f**, and lymphocytes (HE, × 400). **g**–**i**: Cutaneous sarcoidosis. **g** Dense, non-necrotizing granulomas and inflammatory cells in the dermis are accompanied by epidermal hyperkeratosis (HE, × 200). **h** The same image as in G but at higher magnification shows that the granulomas are uniform in size and shape (HE, × 400). **i** The granulomatous infiltrate can occasionally extend from the dermis to the subcutaneous adipose tissue (HE, × 400)

Typical non-necrotizing epithelioid granulomas were seen in lung, lymph node or skin biopsies (Fig. 2a–i). The granulomas, consisting of epithelioid cells, giant cells and lymphocytes, exhibited a lymphatic pattern that surrounded the bronchovascular structures; angioinvasion was seen in some cases as well.

Ocular manifestations were detected in 33 of our 115 patients with SS (29%). The involvement was bilateral in 29 patients (88%) and the presenting feature in 13 of these patients (39%). The diagnostic procedures carried out in the 33 patients are summarized in Table 1. The First International Workshop on Ocular Sarcoidosis for the Standardization of Uveitis Nomenclature (SUN) published diagnostic criteria [28], which were later revised in a consensus workshop [29]. Based on these criteria, definite biopsy-supported ocular sarcoidosis was diagnosed in 32 of the 33 patients (97%). In the remaining patient, in whom no biopsy was performed, the diagnosis of ocular sarcoidosis was supported by an ophthalmologic examination indicating posterior uveitis, chest X-ray and CT scans showing bilateral hilar lymphadenopathy, parenchymal nodules and ground glass opacities, and BAL fluid with CD4 lymphocytosis of 18% and a CD4/CD8 ratio of 4:1.

**Fig. 3:**
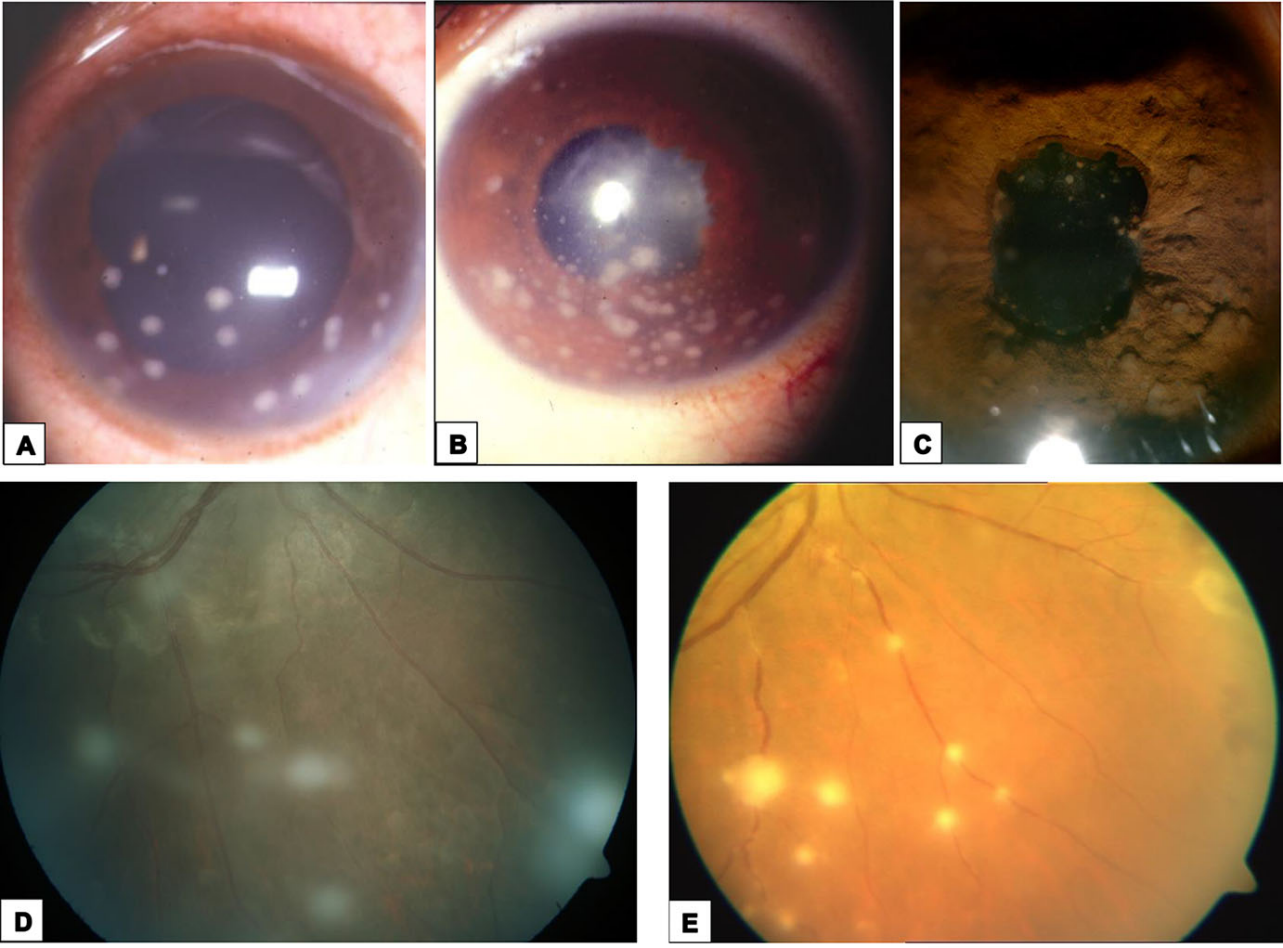
Sarcoidosis-related uveitis. **a** Slit-lamp photograph from a patient with granulomatous anterior uveitis due to sarcoidosis shows large mutton-fat keratic precipitates. **b** In another patient, smaller granulomatous keratic precipitates are seen. **c** Multiple Busacca nodules are clearly recognizable in this slit-lamp photograph from a patient with sarcoid anterior uveitis. **d** Fundus photograph from a patient with sarcoid intermediate uveitis shows snowball opacities. **e** Sarcoid retinal vasculitis with candle wax drippings

Among the variable patterns of ocular sarcoidosis, eyelid and orbital involvement as well as tarsal and bulbar granulomas were uncommon findings. By contrast, anterior chronic granulomatous uveitis was the most frequent ophthalmologic manifestation and was diagnosed in 12 patients (36%), with bilateral involvement in 11 of them. Ocular pain, redness, photophobia, and lachrymation of variable severity were present in 9 patients, whereas the remaining 3 patients had a mild, almost silent uveitis. The ophthalmologic examination of the 12 patients revealed stellate keratic precipitates of the large mutton-fat-type (*n* = 8 patients, Fig. 3a, b), Koeppe nodules on the pupillary border (*n* = 3 patients) and Busacca nodules of the stroma (*n* = 1 patient, Fig. 3c). Posterior synechiae, glaucoma, and cataract as complications were observed in 4, 2, and 3 patients, respectively.

Anterior uveitis was also diagnosed in a 34-year-old male patient with a clinically distinct phenotype of sarcoidosis called Löfgren syndrome, which is characterized by acute-onset and recurrent erythema nodosum, acute iridocyclitis, bilateral hilar adenopathy and migratory polyarthritis [30]. In this patient, steroid-induced remission was achieved in just over one year. In a 41-year-old female patient who complained of dry eyes and mouth, anterior uveitis was associated with low-grade fever, bilateral parotid gland enlargement, and right facial nerve palsy. She was diagnosed with uveoparotid fever (Heerfordt syndrome) [31]. A therapeutic daily regimen of 0.5 mg prednisone/kg body weight with progressive tapering resulted in complete remission within 3 months and no further evidence of the disease over the next year.

Intermediate uveitis was detected in 3 patients (9.1%), in whom blurred vision and worsening floaters were common complaints. Slit lamp examination in the first patient, who had been diagnosed with pars planitis, showed several inflammatory cells suspended in the vitreous. In the other two patients, a higher number of cellular aggregates commonly termed “snowballs” (Fig. 3d) were identified in the vitreous, which were associated with vascular leakage on fluorescein angiography. Cataract and macular edema were observed as late complications in all three patients.

Posterior uveitis was diagnosed in 7 patients (21%) who complained of worsening vision, visual field disturbances, and floaters but who had no evidence of central nervous system involvement. On funduscopic examination, four patients had the typical fundus findings known as “candle-wax drippings” (Fig. 3e), that result from segmental retinal periphlebitis with perivenous exudation. Capillary obstruction with vitreous hemorrhage was detected in 2 patients and multifocal chorioretinitis with choroidal granulomas was observed in the seventh patient.

Finally, panuveitis with inflammation of all uveal components was recorded in 9 patients (27%). The clinical presentation included a variable combination of symptoms including redness and photophobia, ocular pain, lachrymation, blurring of vision, floaters, reduction of visual acuity, and photopsia. Fluorescein angiography revealed multiple chorioretinal peripheral lesions in 4 patients and multiple choroidal granulomas in the posterior pole associated with peripheral chorioretinal scars in 3 patients. The remaining 2 patients had only mild ocular complaints at the time of the first ophthalmological examination, despite macular edema, faint vitreous hemorrhage, and increased intraocular pressure (IOP).

Table 2 compares the basic demographic data, laboratory signs, disease phenotype [32], number of organs involved and the medical treatment of patients with (group A) and without (group B) ocular manifestations at diagnosis. Mean age was significantly higher in group B than in group A patients. Apart from ocular disease, which was the distinguishing feature in all group A patients but by definition absent in group B patients, the lungs and thoracic lymph nodes were the most frequently involved sites in both groups. The frequency of skin, peripheral lymph node, liver, spleen, heart and kidney involvement did not significantly differ between the two groups. There was also no significant difference in the number of group A and group B patients in whom from 1 to ≥ 4 organs were determined to be involved at the time of diagnosis.

**Table 2:** Comparison between the patients with (group A) and without (group B) ocular manifestations at diagnosis

	Group A 33 patients (%)	Group B 82 patients (%)	*p*-value
Males	12 (36)	36 (44)	0.4603
Females	21 (64)	46 (56)	
M/F ratio	1/1.8	1/1.3	
Mean age ± standard deviation (years)	53.3 ± 9.64	59.5 ± 8.43	0.0009
ACE > 70 U/L	25/29 (86)	73/76 (96.1)	0.0719
Calcemia > 11 mg/dL	5/33 (15.2)	14/69 (20.3)	0.5787
Calciuria > 320 mg/24 h	9/27 (33.3)	19/47 (40.4)	0.5475
Organs involved, no. of patients (%)			
Eyes(including 13 patients with ocular symptoms as presenting feature)	33 (100)	–	< 0.001
Lungs and thoracic lymph nodes	17 (51.5)	76 (92.7)	< 0.001
Skin (including erythema nodosum)	4 (12.1)	19 (23.1)	0.209
Peripheral lymph nodes	3 (9.1)	17 (20.7)	0.178
Liver	3 (9.1)	11 (13.4)	0.754
Spleen	–	6 (7.3)	0.180
Heart	3 (9.1)	4 (4.9)	0.407
Kidney	–	3 (3.7)	0.556
No. of organs involved in each patient (%)			
1 organ	13 (39.4)	40 (48.8)	0.361
2 organs	14 (42.4)	33 (40.3)	0.829
3 organs	4 (12.1)	8 (9.7)	0.741
≥ 4 organs	2 (6.1)	1 (1.2)	0.197
Medical treatment			
Topical CS (eyedrops, sub-Tenon’s injections, intravitreal implants)	18	–	
Systemic CS alone	7	16	
CS plus Cyclophosphamide	–	9	
CS plus Azathioprine	–	13	
CS plus Methotrexate plus the later addition of Azathioprine	10	22	
CS plus Adalimumab	6	13	
CS plus Methotrexate plus Rituximab	–	9	

The mean duration of follow-up was 48.8 ± 19.2 months (range, 13–84). A later appearance of ocular disease was recorded in 5 group B patients during follow-up: 2 patients developed posterior uveitis 15 and 18 months after the diagnosis whereas in the other 3 patients panuveitis was detected approximately 24, 31 and 36 months after the initial diagnosis. In all five patients, the ocular manifestations were diagnosed 6 weeks to 4 months after the discontinuation of CS treatment (3 patients) or during tapering to a maintenance dose (2 patients). Seven group A patients who had lung involvement at the time of diagnosis experienced pulmonary exacerbations within 1–2 years after the initial diagnosis. Three additional group A patients developed liver and heart involvement within 2 years of diagnosis, while still receiving low doses of CS.

## Treatment

Given the persistent mystery regarding the etiology of sarcoidosis, its first-line treatment continues to be based on non-specific agents. Topical CS eyedrops, mostly prednisolone acetate 1% with variable frequency of administration (usually four times daily), were used to treat our patients with anterior uveitis, with the exception of two patients who instead received rimexolone 1% eyedrops. A mild increase in the IOP but no development of glaucoma was observed in three patients. CS eyedrops were combined with cycloplegics to relieve ciliary muscle spasm and to prevent the formation of posterior synechiae.

Of the three patients with intermediate uveitis, one was treated with sub-Tenon’s injections of triamcinolone acetonide, whereas the other two patients received a dexamethasone intravitreal implant (Ozurdex®, Allergan Pharmaceuticals) that was replaced every 5–6 months. The same type of intravitreal implant was employed in 3 and 4 patients with posterior uveitis and panuveitis, respectively. An increased IOP was a common complication and resulted in glaucoma and cataract in 3 patients.

**Fig. 4:**
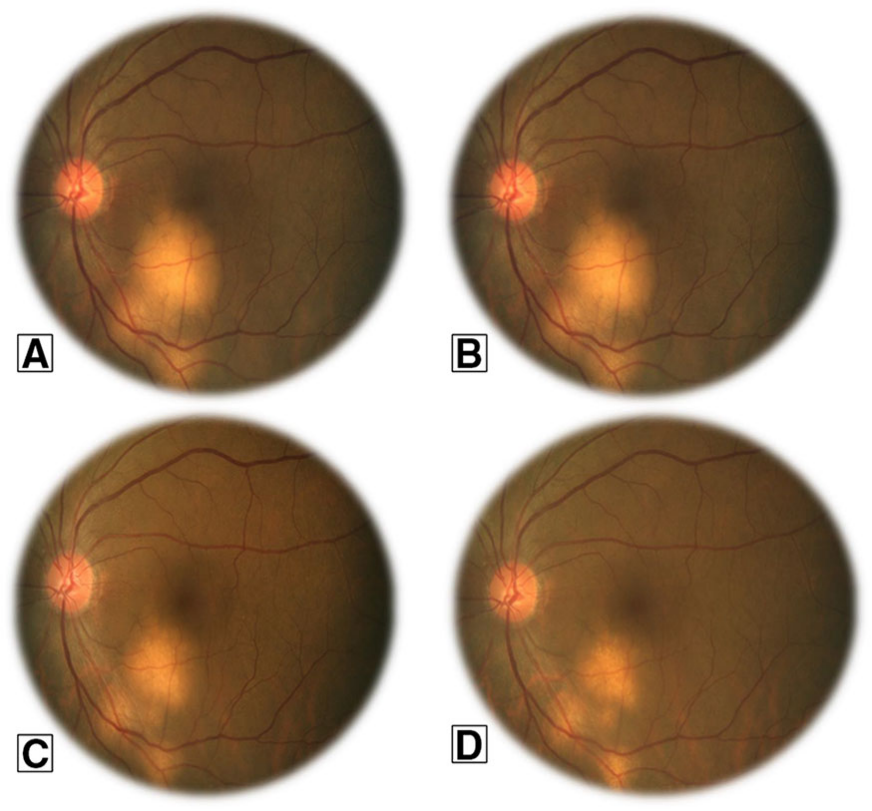
Choroidal sarcoidosis and the response to corticosteroids. A fundus photograph of the left eye of a 65-year-old man with systemic sarcoidosis shows multiple choroidal nodules. The patient was started on a daily regimen of 1 mg oral prednisone/kg body weight. Serial fundus photographs after the initiation of treatment reveal a gradual reduction in the size of the nodules with scarring

Twenty-three patients, treated or not with topical CS, were administered systemic (invariably oral) CS. The initial dose of 0.5–1 mg/kg/day was gradually tapered according to the clinical course while maintaining the best balance between efficacy and side-effects (Fig. 4). The decision to switch from topical CS eyedrops or intravitreal CS implants to the systemic administration of CS, with or without steroid-sparing agents, was based on both the persistent severity of the uveitic disease and the concomitant occurrence of extra-ocular manifestations, especially respiratory tract involvement. Following the initiation of systemic treatment, none of the patients required additional Ozurdex implants.

Although CS remains the first-line treatment in the large majority of sarcoidosis patients, clear evidence of its long-term benefits from randomized controlled studies is lacking [33]. In addition, CS-related complications such as hypertension, hyperglycemia, Cushing-like syndrome, and osteoporosis, alone or in combination, develop in the majority of patients.

A wide variety of steroid-sparing agents, including azathioprine, methotrexate (MTX), leflunomide, and mycophenolate mofetil, are commonly used as second-line therapy in patients with refractory or relapsing SS and in those who suffer serious adverse effects from CS [33–35]. In our series, MTX was the most commonly employed immunosuppressive agent and, in combination with CS, was administered at a dose of 25 mg weekly to the 4 and 6 patients with posterior uveitis and panuveitis, respectively. In these same patients, in step with progressive tapering of the CS to a daily maintenance dose of 0.1–0.2 mg/kg and the concomitant reduction of MTX to 15 mg weekly, a low-dose of the antimetabolite azathioprine (50 mg daily) was added to the therapeutic regimen. Overall, satisfactory results were achieved in all patients in terms of both efficacy and safety. None of the patients were treated with mycophenolate mofetil, whose efficacy in a comparative randomized clinical trial was not higher than that of MTX; on the contrary, treatment success was 22% higher with MTX [36].

Three patients with posterior uveitis and 3 with panuveitis received a combination of gradually tapered CS (from 0.5 to 0.1 mg/kg daily) plus adalimumab (40 mg subcutaneously every 2 weeks for 12 weeks). These patients had concomitant lung involvement (pulmonary infiltration with or without lymphadenopathy [37], with a decreased diffusing lung capacity for carbon monoxide (DLCO), and had experienced frequent relapses of their ocular manifestations during stepwise reductions in the doses of CS and MTX. In 5 of the 6 patients treated with tapered CS and adalimumab, uveitic flares did not occur throughout the 12-month period of post-treatment follow-up. In all 5 patients, mean levels of C-reactive protein and ACE significantly decreased and DLCO improved, albeit to a variable extent. In the sixth patient, panuveitis worsened by the end of CS plus adalimumab treatment and a full-dose regimen of 1 mg CS/kg daily combined with 25 mg weekly of MTX was therefore resumed. All six patients administered adalimumab reported side effects of injection-site allergic reaction, fatigue, and malaise.

The overall visual outcome in both groups was satisfactory, although complete follow-up data were available only for the first 2 years and were fragmentary thereafter. Of the 62 eyes affected by sarcoid uveitis at diagnosis, the best corrected visual acuity (BCVA) was 20/20 or better in 24 eyes (38.7%), not worse than 20/50 in 36 eyes (58.1%) and reduced to counting fingers in the remaining two eyes. After one year, 29 eyes (46.8%) had a VA of 20/20 or better; three eyes lost 2 lines of VA and the VA of the two eyes with vision reduced to counting fingers remained unchanged. According to our last reliable VA data, recorded 2 years from the time of diagnosis, there was a further improvement in 37 (59.7%) of the eyes, with a VA of 20/20 or better. However, there was no recovery in the eyes that had lost 2 lines of VA and in those with counting fingers vision. Neither bilateral severe visual impairment nor blindness occurred in any of our patients.

Ocular surgery became inevitable in 16 patients (48%) and consisted of cataract extraction in 7 patients, trabeculectomy in 5 patients, and retinal pigment epithelial detachment (RPED) in 4 patients. One of the RPED patients had anterior granulomatous uveitis; the detachment was ascribed to a choriocapillary inflammatory process. The other 3 patients had panuveitis. In one of these patients, a rapid reduction in the size of the choroidal granulomas following CS treatment may have favored a tear in the RPE whereas in the other 2 patients budding during choroidal neovascularization through Bruch’s membrane was hypothesized as the underlying mechanism of serous RPED.

One patient with posterior uveitis became legally blind in her right eye after developing cystoid macular edema.

## Discussion

The diagnosis of ocular sarcoidosis in our cohort was firmly established on the basis of typical ophthalmological features, radiologic findings and histological evidence of non-caseating epithelioid granulomas in biopsy specimens from ocular and/or extra-ocular tissue. An increased proportion of CD4 + helper T-cells with an elevated CD4/CD8 ratio in BAL fluid, detected in a high proportion of our patients (Table 1), has a specificity of ~ 95% for sarcoidosis, although the sensitivity is slightly below 60% [21]. In these cases, the diagnosis is further supported by the additional detection of high serum ACE levels [38] and by the presence of dysregulated calcium homeostasis. Hypercalcemia was detected in 15% of our patients at diagnosis. It has been reported with a similarly low prevalence (5–11%) in other studies [25–27], although it may be missed in those with subacute sarcoidosis, given the frequently undulating course of this form of the disease. Hypercalciuria, shown to be three times more frequent than hypercalcemia in SS [26], was determined in 41% of our patients.

Both literature reports and our own experience consistently evidence an association of chronic SS with a wide spectrum of ocular inflammatory manifestations, detected in 29% of our patients and occurring as the presenting feature in over one-third of them. Any segment of the eye and its adnexa may be affected, but uveitis is the most frequently diagnosed condition [11–13, 39]. The prevalence of uveitis as the initial presenting complaint of SS ranges from 20–30% [14, 16] to almost 80% [11], depending on the population studied and the length of ophthalmic follow-up. Conversely, 30–60% [13] and up to 80% [12, 15, 16] of patients with SS may develop ophthalmic disease at some point during the course of the disease.

In our cohort, the anatomical types of intraocular inflammation were, in descending order of frequency, anterior uveitis, panuveitis, and posterior and intermediate uveitis. At variance from studies in which the majority of patients were Black, posterior and anterior uveitis is the most common localization of sarcoid uveitis in Caucasian patients (reviewed in [12]). Depending on disease severity and the timing of therapy initiation, sarcoid uveitis remains a potentially sight-threatening condition that can cause permanent visual deterioration of variable degree [14, 40]. In our patients, a reliable estimation of the impact of sarcoid uveitis on visual function was restricted to the first 2 years of follow-up, when, compared with the BCVA at diagnosis, a VA of 20/20 or better was recorded in the large majority of the affected eyes. However, no improvement was found in the eyes that had lost two lines of VA one year after diagnosis and in those with counting fingers vision. Irreversible visual loss is most frequently associated with posterior uveitis complicated by cystoid macular edema, which occurred in one of our patients, but ocular hypertension and cataract formation may also lead to visual loss, albeit with a lower frequency [13, 14].

Pulmonary involvement is the most common extraocular manifestation of SS, occurring in > 90% of patients, with consequent respiratory symptoms and abnormal lung function [5, 10, 41]. The comparative analysis of our patients with (group A) and without (group B) ocular manifestations at diagnosis showed that patients in group A were significantly younger and had significantly less pulmonary involvement than those in group B (Table 2), suggesting that ocular sarcoidosis has its own specificity. However, the measured values of ACE, calcemia and calciuria were not significantly different between the two groups. In addition, the drugs used in treatment were fairly variable across the two groups and the outcomes only partially comparable. One possible explanation for the lower prevalence of lung involvement in group A patients is that the occurrence of eye symptoms compels the patient to seek a prompt ophthalmological examination, which usually results in an earlier diagnosis. With the initiation of a suitable therapy, involvement of the pulmonary parenchyma and mediastinal lymph nodes may be delayed or even prevented. Alternatively, considering that all our patients were ethnically homogeneous Caucasians, the differences in the prevalence patterns of the individual organ manifestations may reflect environmental factors [1, 42].

**Fig. 5:**
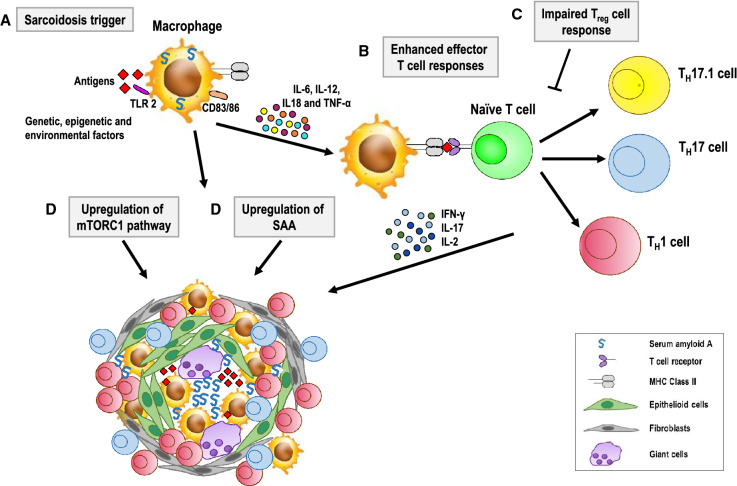
Hypothetical pathogenetic pathway of sarcoidosis. Antigens interact with genetic, epigenetic and environmental factors to cause the following: **a** TLR2-mediated macrophage activation with the production of pro-inflammatory cytokines, such as TNF-α, IL-12, IL-18 and IL-6; **b** antigen presentation by MHC class II molecules on macrophages to CD4^+^ T cells; activation and differentiation of CD4^+^ T cells into Th1 and Th17.1 effector cells that produce IFN-γ and IL-2, and Th17 cells that produce IL-17; **c** impaired regulatory T cell response; **d** up-regulation of the mTORC1 pathway; **e** up-regulation of SAA followed by SAA aggregation within the granuloma and amplification of the Th1 immune response. Abbreviations. TLR: toll-like receptor; MHC: major histocompatibility complex; IL: interleukin; TNF: tumor necrosis factor; IFN: interferon; mTORC1: mechanistic target of rapamycin complex 1; SAA: serum amyloid A

Although the etiology of sarcoidosis is unknown, a better understanding of the pathogenetic mechanisms underlying its onset is clearly important for the development of a more rational and effective therapeutic approach. The basic mechanism that triggers and maintains the inflammatory state is an interaction between lymphocytes and macrophages within the granulomatous areas, with the compartmentalization of T-cells expressing the helper (Th) phenotype [43]. Immunologically, sarcoidosis is characterized by an exaggerated Th type 1 (Th1) immune response to a so far unknown antigen(s) (Fig. 5). The interaction between antigens and innate immune receptors, such as Toll-like receptor 2, induces the activation of macrophages which then become highly efficient antigen-presenting cells able to interact with T cells. Activated macrophages also release pro-inflammatory cytokines, including tumor necrosis factor (TNF)-α, a central mediator in the initiation and amplification of the granulomatous response, as well as interleukin (IL)-12, IL-18 and IL-6, which modulate the type of T cell response [44, 45]. The levels of chemokines and chemokine receptors associated with a Th1 response are also increased [46] and, together with pro-inflammatory cytokines, orchestrate granuloma formation.

Topical, regional, and systemic CS remain the mainstay of therapy for chronic ocular sarcoidosis. Steroid-sparing immunosuppressive agents, which in our cohort consisted of MTX and azathioprine, may be required in patients who are or become dependent on, unresponsive to or poorly tolerant of CS treatment. However, for patients with SS not controlled by first- and second-line therapies, the obvious therapeutic choice is a combination of CS and a biologic agent. This was the case in 6 patients of our cohort, who were treated with CS plus adalimumab. All but one of these patients remained free of uveitic flares for one year post-treatment. Successful use of the chimeric antibody infliximab and the fully humanized monoclonal antibody adalimumab in the treatment of severe and refractory chronic sarcoidosis has been reported and both have become established third-line agents [47–49]. These antibodies target TNF-α, which, as mentioned above (Fig. 5), plays a central role in the initiation and amplification of the inflammatory response. In addition to TNF-α inhibitors, a steadily growing list of other biologic agents, such as lymphocyte inhibitors and specific receptor antagonists, are being tested in patients with non-infectious uveitis, including sarcoidosis, both in observational case series and in non-randomized off-label studies [16, 50].

The caution shown by experienced clinicians in recommending systemic biologic agents reflects the high cost of these drugs, the scarcity of randomized clinical trials confirming their efficacy and long-term safety data. Although a significant proportion of patients with systemic autoimmune diseases have been effectively treated with licensed biologic agents as second- or third-line therapies targeting a specific molecular component of the immune system, the paradoxical induction by these drugs of organ-specific or systemic autoimmune processes, including sarcoidosis, has been reported. Perez-Alvarez et al. [51] documented over 1500 cases involving these unexpected events and thus clearly demonstrated that biologics, in spite of their undoubted utility, may be a double-edged sword [52].

Among the strengths of our study are: (1) the diagnosis of systemic sarcoidosis supported by biopsies of involved organs in the large majority of patients; (2) the homogeneous collection of data, made possible through a carefully planned collaboration between an eye-care clinic and a clinical immunology tertiary center of the same university hospital; (3) the length of the patients’ follow-up, which exceeded 4 years in the majority, (*n* = 71, 62%) and (4) clinical and ophthalmological assessments made by the same internists and the same ophthalmologists to avoid or reduce the risk of variability.

The potential shortcomings must be noted as well, including: (1) the retrospective, observational nature of the study; (2) the relatively small number of patients, compared to multicenter, population-based cohort studies [10, 42, 53–55],(3) clinical and biochemical data that were only partially available for 5 of our patients with ocular manifestations and (4) the fact that an ophthalmologic examination was not performed in the first 17 patients diagnosed with systemic sarcoidosis, in whom the occurrence of subclinical or mildly symptomatic ocular manifestations could not, therefore, be excluded.

Patient education and scheduled periodic controls are recommended to achieve a timely diagnosis, to taper or conversely upgrade treatment as required, and to limit—to the extent possible—extraocular damage. A multidisciplinary collaboration among different healthcare providers, including the ophthalmologist, internist, immunologist and rheumatologist, will likely result in a more precise diagnostic workup and a more effective therapeutic strategy for all patients with sarcoidosis. Two important clinical challenges that remain to be met are the recognition of patients at risk of developing progressive ocular and multi-organ disease, and the decision when to stop treatment. Their resolution awaits the results of longitudinal studies with congruous numbers of patients.
